# Spherical multigrid neural operator for improving autoregressive global weather forecasting

**DOI:** 10.1038/s41598-025-96208-y

**Published:** 2025-04-04

**Authors:** Yifan Hu, Fukang Yin, Weimin Zhang, Kaijun Ren, Junqiang Song, Kefeng Deng, Di Zhang

**Affiliations:** 1 College of Computer Science and Technology, National University of Defense Technology, Changsha, 410073 People’s Republic of China; 2 College of Meteorology and Oceanography, National University of Defense Technology, Changsha, 410073 People’s Republic of China

**Keywords:** Spherical convolution, Autoregressive forecasts, Multigrid neural operator, Spherical shallow water equations, Global weather forecasting, Atmospheric science, Atmospheric dynamics

## Abstract

Data-driven approaches for global weather forecasting have shown great potential. However, conventional architectures of these models struggle with spherical distortions, leading to unstable autoregressive forecasts. Although methods such as spherical Fourier neural operator (SFNO) based on spherical harmonic convolution can alleviate these problems, they face the challenge of high computational cost. Here, we introduce a spherical multigrid neural operator (SMgNO) that integrates spherical harmonic convolution and low resolution SFNO in the multigrid framework, effectively alleviating data distortions while requiring few computational resources. Experiments for spherical shallow water equations and medium-range global weather forecasting demonstrate the effectiveness and robustness of SMgNO. For 500 hPa geopotential height with a 7 days lead time, SMgNO achieves a 9.31% and 6.83% improvement in anomaly correlation coefficient over IFS T42 and SFNO, respectively. Furthermore, SMgNO requires only 10% floating-point operations of SFNO for forward propagation and 30.90% less GPU memory than SFNO during training.

## Introduction

Accurate and timely weather forecasting plays an important role in many aspects of human society. Numerical weather prediction (NWP) model is the predominant method for weather forecasting^1,2^, which simulates the future state of the atmosphere by solving the partial differential equations (PDEs) numerically^3^. Although NWP models are capable of producing accurate forecasts, they often exhibit slow processing speeds and require the support of high-performance computing systems^1–4^. Moreover, errors in initial conditions, approximations of physical processes in parameterizations, and the chaos of the atmosphere introduce uncertainties to NWP^1,3^.

Recently, deep learning has significantly transformed the domain of weather forecasting, facilitating the generation of timely forecasts. For instance, Rasp and Thuerey^5^ employed a deep residual convolutional neural network (CNN) known as ResNet^6^ to conduct continuous forecasts at a spatial resolution of 5.625° × 5.625°, achieving performance comparable to that of a physical baseline at a similar resolution. The FourCastNet^7^ model firstly enhanced the resolution of data-driven global weather forecasting to 0.25° × 0.25°, but its accuracy remains slightly inferior to that of the most advanced NWP system (the operational integrated forecasting system (IFS) of the European Centre for Medium-Range Weather Forecasts (ECMWF)). Before long, data-driven weather forecasting system have made significant advancements. For example, Pangu-Weather^4^ produces stronger deterministic forecast results than the operational IFS on many tested weather variables. Shortly thereafter, GraphCast^2^ outperformed the IFS on a broader range of variables and exhibited enhanced capabilities in predicting severe weather events. In 2023, a variant of the vision transformer known as FengWu^8^ addressed the medium-range forecasting challenge from a multi-modal and multi-task perspective, achieving state-of-the-art performance for long-term forecast lead times. Furthermore, FuXi^1^ was published with performance comparable to that of the ECMWF ensemble mean (EM) in 15 days forecasts.

However, the errors of data-driven models tend to accumulate rapidly during autoregressive global forecasting. To address this issue, researchers have explored various strategies to mitigate error accumulation. Two popular methods include combining multiple models to reduce the number of autoregressive steps and employing fine-tuning techniques to enhance multi-step forecasts. For example, Pangu-Weather^4^ trained four models across different lead times and employed a greedy hierarchical temporal aggregation strategy to minimize the number of autoregressive steps. Similarly, Fuxi^1^ optimized performance for both short and long lead times by utilizing a cascade^9,10^ model architecture and fine-tuning the pre-trained models within specific 5 days forecast time windows. Furthermore, FengWu^8^ mitigated the intermediate input error during the autoregressive inference stage by using a replay buffer to store the predicted results from previous optimization iterations, which were then utilized as input for the current model.

Despite these approaches have yielded promising results, the models fail to account for the fact that the data are situated on a sphere, which leads to distortions. These distortions negatively impact the performance of autoregressive forecasts. To address these distortions and improve autoregressive global weather forecasting, some researchers have modified the model architecture based on prior knowledge, making it more suitable for predicting spherical dynamical processes. For instance, Weyn et al.^11^ introduced cubed-sphere remapping, which minimizes distortion on the cube faces and provides natural padding for convolution operations. Due to the improvements in long-term predictions brought by this cubed-sphere grid, Weyn et al.^12^ further employed this framework along with large multi-model ensemble techniques for sub-seasonal forecasting. Following Weyn et al.^11^, Lopez-Gomez et al.^13^ utilized the U-Net 3+ architecture^14^ on this cubed-sphere grid to generate forecasts of extreme surface temperatures. Although, achieving notable improvements, the cubed-sphere grid still suffers data distortion at high latitudes. Subsequently, McCabe et al.^15^ applied the double Fourier sphere (DFS) method to rectify the artificial discontinuities caused by the two-dimensional fast Fourier transform, resulting in reduced errors in long-term forecasting. However, the DFS method still introduces spatial distortions, a drawback that is not present when utilizing spherical harmonic basis functions. The spherical harmonic basis has isotropy and rotation invariance, using spherical harmonic transform (SHT) to process spherical data has natural advantages. To this end, Bonev et al.^16^ introduced the spherical Fourier neural operator (SFNO) based on SHT, demonstrating stable autoregression while maintaining physically plausible dynamics. Despite the improvement made by SFNO, it increases the amount of computation and requires more training resources.

To address these challenges, we introduce the spherical multigrid neural operator (SMgNO), which is based on both the MgNO^17^ and the SFNO^16^. The SMgNO employs convolutions based on spherical harmonic functions (CSHFs), similar to SFNO^16^, to mitigate distortions and ensure the stability of autoregressive global forecasting. Furthermore, inspired by MgNO^17^, SMgNO utilizes the multigrid framework instead of the Transformer framework to reduce computational costs. Experiments for shallow water equations (SWEs) and medium-range global weather forecasting demonstrate the effectiveness and robustness of the proposed methods. The contributions of this work are summarized as follows:

- The instability produced by data distortions in data-driven global weather forecasting models is analyzed via experiments.
- Based on the MgNO^17^ and the SFNO^16^, we propose SMgNO, a novel data-driven weather forecasting framework that improves autoregressive forecast accuracy while reducing computational costs.
- Experiments on spherical SWEs solving and medium-range global weather forecasting demonstrate the effectiveness of the proposed framework.

The subsequent sections of this paper are organized as follows. Section “Methods” provides a succinct overview of the CSHFs and introduces the proposed SMgNO. Section “Data and experiments’’ details the datasets utilized and the experimental designs. Section “Results” describes some universal factors that lead to instability of autoregressive data-driven models and demonstrates the superior performance of the proposed SMgNO by spherical SWEs solving and medium-range global weather forecasting. Finally, Section “Discussion” concludes this work.

## Methods

### Learnable convolutions based on spherical harmonic functions

Let $g\left(\lambda ,\varphi \right)$ and $k\left(\lambda ,\varphi \right)$ be two real-valued functions defined on the unit sphere ${{\varvec{S}}}^{2}$, where $\lambda \in \left[-\pi , \pi \right]$ is the longitude, $\varphi \in [0, \pi ]$ is the colatitude. Then their convolution is defined as an integral over the three-dimensional rotation group $SO(3)$^16,18^:

1 $$ \left( {k * g} \right)\left( {\lambda ,\varphi } \right) = \int_{R \in SO\left( 3 \right)} {k\left( {{\varvec{R}}n} \right) \cdot g\left( {{\varvec{R}}^{ - 1} \cdot \left( {\lambda ,\varphi } \right)} \right)d{\varvec{R}},} $$

where $*$ represents convolution operation, $n$ is the north pole, and ${\varvec{R}}$ is the rotation to the north pole. According to the convolution theorem^19^, this convolution is equivalent to pointwise multiplication of harmonic coefficients:

2 $$ \left( {k*g} \right)\left( {\lambda ,\varphi } \right) = {\mathcal{F}}^{ - 1} \left( {C\left( l \right){\mathcal{F}}\left( g \right)\left( {l,m} \right) \cdot {\mathcal{F}}\left( k \right)\left( {l,0} \right)} \right), $$

where $\mathcal{F},{\mathcal{F}}^{-1}$ are the SHT and inverse spherical harmonic transform (ISHT), $C\left(l\right)=2\pi \sqrt{\frac{4\pi }{2l+1}}$, $l,m$ are the degree and order of harmonic functions, respectively. The learnable CSHFs was derived by substituting the filter kernel $C(l)\mathcal{F}\left(k\right)\left(l,0\right)$ in Eq. (2) with a learnable kernel ${\widetilde{k}}_{\theta }(l)$:

3 $$ \left( {k_{\theta } * g} \right)\left( {\lambda ,\varphi } \right) = {\mathcal{F}}^{ - 1} \left( {{\mathcal{F}}\left( g \right)\left( {l,m} \right) \cdot \tilde{k}_{\theta } \left( l \right)} \right). $$

The spherical harmonic expansion of function $g\left(\lambda ,\varphi \right)$ is expressed as follows:

4 $$ g\left( {\lambda ,\varphi } \right) = \mathop \sum \limits_{l = 0}^{\infty } \mathop \sum \limits_{m = - l}^{l} \hat{g}_{lm} \cdot Y_{lm} \left( {\lambda ,\varphi } \right), $$

where

5 $$ \hat{g}_{lm} = \int_{{S^{2} }} {g\left( {\lambda ,\varphi } \right) \cdot Y_{lm} \left( {\lambda ,\varphi } \right)d\lambda \,d\varphi ,} $$

is the associated coefficient of spherical harmonics ${Y}_{lm}$. Consequently, the formulation of the learnable CSHFs is presented as follows:

6 $$ \left( {k_{\theta } * g} \right)\left( {\lambda ,\varphi } \right) = \mathop \sum \limits_{l = 0}^{\infty } \mathop \sum \limits_{m = - l}^{l} \hat{g}_{lm} \cdot \tilde{k}_{\theta } \left( l \right) \cdot Y_{lm} \left( {\lambda ,\varphi } \right). $$

In practical implementations, it is often necessary to truncate the spherical harmonic expansion at a specific bandwidth $L$, which inevitably introduces truncation error. To address this problem, Ha and Lyu^18^ theoretically demonstrated that the truncated high-frequency information could be approximated by a scaled ISHT of ${\widehat{g}}_{lm}$. As a result, Eq. (6) can be reformulated as follows^18^:

7 $$ \left( {k_{\theta } * g} \right)\left( {\lambda ,\varphi } \right) \approx \mathop \sum \limits_{l = 0}^{L} \,\mathop \sum \limits_{m = - l}^{l} \hat{g}_{lm} \cdot \tilde{k}_{\theta }^{\prime } \left( l \right) \cdot Y_{lm} \left( {\lambda ,\varphi } \right) + \alpha g\left( {\lambda ,\varphi } \right). $$

where $\tilde{k}_{\theta }^{\prime } = \tilde{k}_{\theta } - \frac{\alpha }{C\left( l \right)}$, $\alpha$ is a learnable parameter, and $\alpha g\left(\lambda ,\varphi \right)$ represents an impulse response at the north pole. For a detailed derivation, please refer to Ha and Lyu^18^.

### Architecture of the SMgNO

The multigrid method^20^ is recognized as one of the effective numerical techniques for solving PDEs. Inspired by this method, He et al.^17^ introduced the MgNO, which achieved state-of-the-art performance in solving a variety of PDEs by parameterizing linear operators among neurons through multigrid structures. In this section, we will first provide a succinct overview of the multigrid method and the MgNO. Subsequently, we will introduce the SMgNO, which is based on both the MgNO^17^ and the SFNO^16^.

The pseudo-code for the multigrid method utilizing a V-cycle to solve the linear system $Au = f$ is presented in the supplementary information, where $A$ is the system operator, $u$ is the variables, and $f$ is the right-hand side of the equation. The primary components of the multigrid method can be categorized into the system operator $A$, smoothing operator $S$, restriction operator $R$, prolongation operator $P$, and solvers on the coarse grids. The MgNO^17^ framework employs the convolutions with a 3 × 3 kernel size and a 1 × 1 stride size to parameterize the system operator and smoothing operator. Additionally, it utilizes convolutions with a 3 × 3 kernel size and a 2 × 2 stride size to parameterize the restriction operator, while the prolongation operator is parameterized using a transposed convolution operator with a 4 × 4 kernel size and a 2 × 2 stride size.

**Fig. 1:**
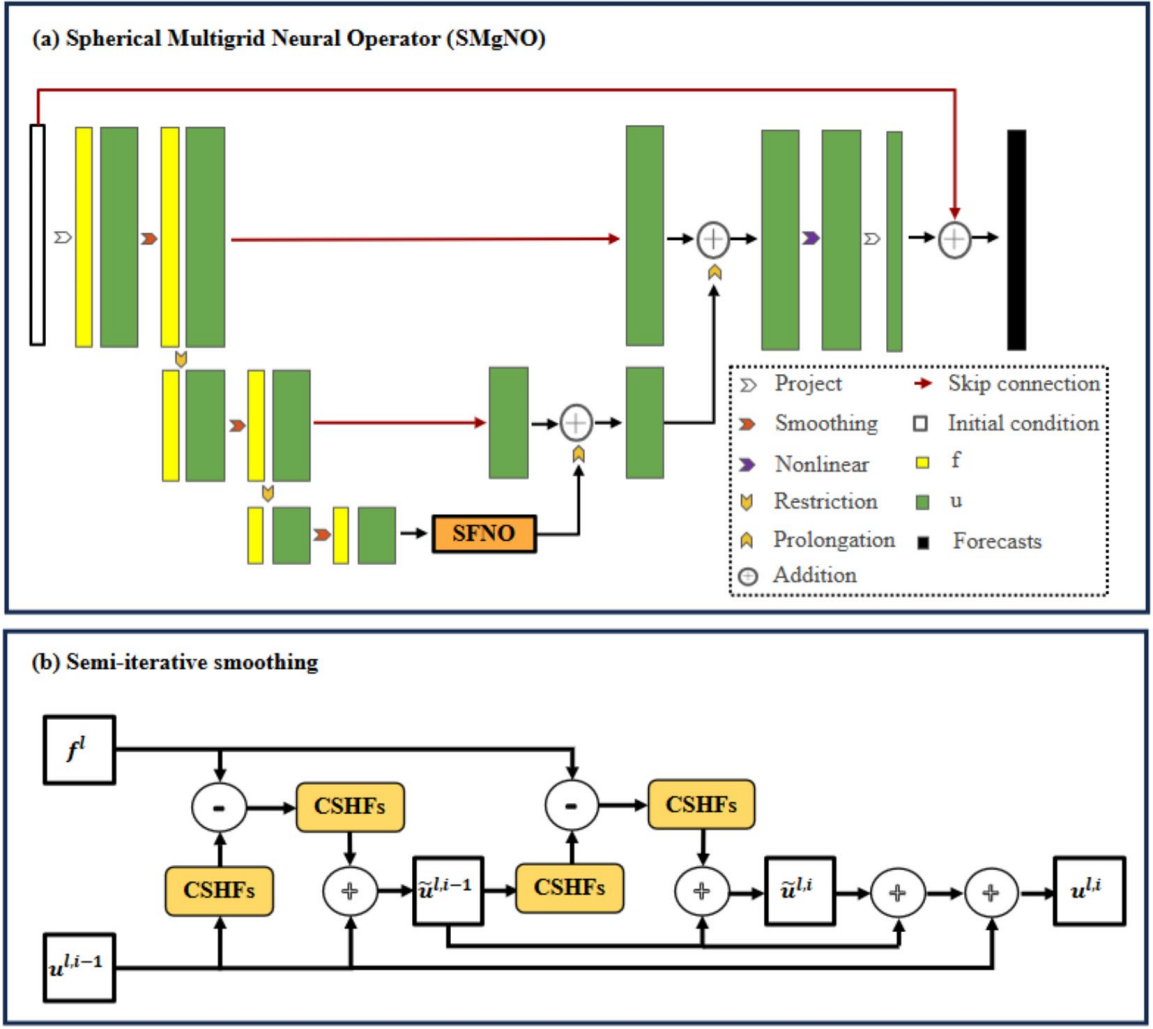
(**a**) Overview of the SMgNO architecture; (**b**) The semi-iterative smoothing operation. In the Figure, “CSHFs” is short for “convolutions based on spherical harmonic functions”.

To address the issue of spherical data distortion, we implement the CSHFs described in Eq. (7) for the system operator and smoothing operator. We retain the same restriction operator as employed in MgNO^17^, while using periodic padding in the longitudinal direction to ensure continuity. Given that transposed convolution may lead to checkerboard artifacts^21^, we substitute it with pixel shuffle operations^22^. In the coarsest grid, however, the MgNO solely utilizes the smoothing mechanism without integrating an underlying solver. Consequently, as illustrated in Fig. 1a, we incorporate the SFNO^16^ into the coarsest grid to enhance the accuracy of the solutions at this level. Furthermore, drawing upon the methodologies of LM-ResNet^23^ and MgNet^24^, we have modified the residual correction smoothing technique (lines 5–8 in Algorithm S1) to a semi-iterative smoothing approach (see Fig. 1b and lines 5–9 in Algorithm 1). The framework of SMgNO is described in Algorithm 1, where ${u}^{l, i}$ denotes the feature at grid level $l$ that has undergone smoothing $i$ times, ${A}^{l}$ and ${S}^{l}$ are the discretions of the system operator and smoothing operator, ${R}_{l}^{l + 1}$ and ${\Pi }_{l}^{l + 1}$ are restriction operators which transfer the feature from the fine grid (level $l$) to the coarse grid (level $l+1$), ${P}_{l + 1}^{l}$ is prolongation operator that converts the feature from the coarse grid (level $l+1$) to the fine grid (level $l$), ${k}^{l}$ is a learnable parameter, $W$ is a learnable projection matrix, and $\sigma$ is a point-wise Gaussian error linear unit^25^, $L$ and ${v}_{l}$ are constants, representing the maximum grid level and smoothing times, respectively.

**Algorithm 1:**
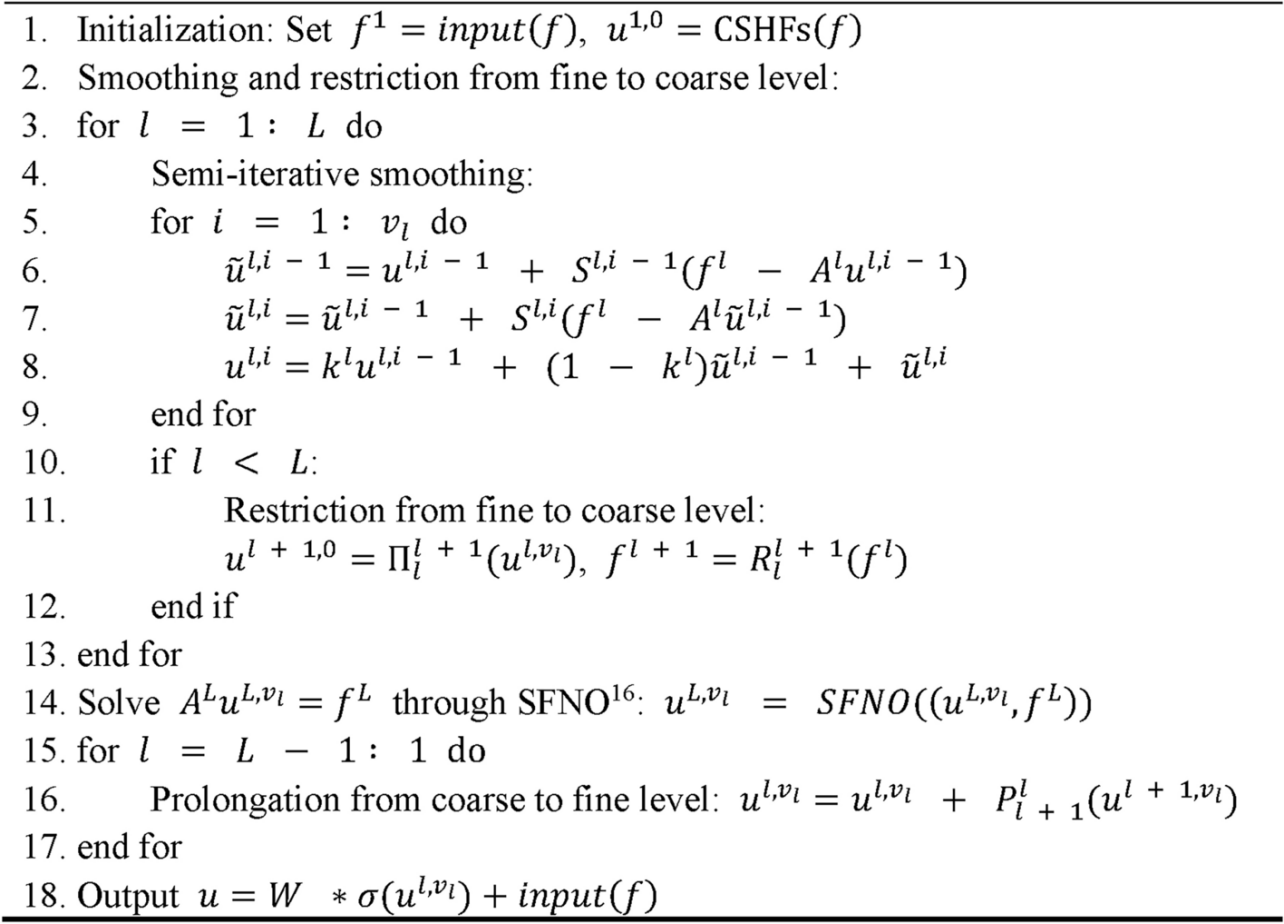
S*MgNO*(*u,f*). The spherical multigrid neural operator with V-cycle.

## Data and experiments

### Spherical shallow water equations

The SWEs on rotating sphere are a nonlinear hyperbolic PDEs system^16^. They are derived by integrating the Navier–Stokes equations over the depth of the fluid layer when the horizontal length scale is much larger than the vertical length scale. They are formulated as follows:

8 $$ \left\{ {\begin{array}{*{20}l} {\frac{\partial \varsigma }{{\partial t}}{ } = { } - \frac{1}{{a\,{\text{cos}}\theta }}\frac{\partial }{\partial \lambda }\left[ {\left( {\varsigma + { }f} \right)u} \right]{ } - { }\frac{1}{{a\,{\text{cos}}\theta }}\frac{\partial }{\partial \theta }\left[ {\left( {\varsigma + { }f} \right)v\,{\text{cos}}\theta } \right] ,} \hfill \\ {\frac{\partial \delta }{{\partial t}}{ } = { }\frac{1}{{a\,{\text{cos}}\theta }}\frac{\partial }{\partial \lambda }\left[ {\left( {\varsigma + { }f} \right)v} \right]{ } - { }\frac{1}{{a\,{\text{cos}}\theta }}\frac{\partial }{\partial \theta }\left[ {\left( {\varsigma + { }f} \right)u\,{\text{cos}}\theta } \right]{ } - { }\nabla^{2} \left[ {\varphi + { }\frac{1}{2}\left( {u^{2} { } + { }v^{2} } \right)} \right] ,} \hfill \\ {\frac{\partial \varphi }{{\partial t}}{ } = { } - \frac{1}{{a\,{\text{cos}}\theta }}\frac{{\partial \left( {\varphi u} \right)}}{\partial \lambda }{ } - { }\frac{1}{{a\,{\text{cos}}\theta }}\frac{{\partial \left( {\varphi v\,{\text{cos}}\theta } \right)}}{\partial \theta }{ } - \overline{\varphi }\delta .} \hfill \\ \end{array} } \right. $$

where $f = 2\Omega \,\sin \theta$ is the Coriolis parameter with ${\Omega }$ being the angular velocity of the sphere, $\varsigma ,{ }\delta ,{ }\varphi ,{ }\overline{\varphi }, u, v,{ }a$ are vorticity, divergence, geopotential height, mean geopotential height, the λ- and the θ-components of the velocity vector in the spherical coordinates, and the radius of the sphere, respectively. As a simplification of the fluid motion equations, the SWEs are extensively utilized in various fields, including atmospheric dynamics, tidal motion, tsunami propagation, and the simulation of Rossby and Kelvin waves. The accuracy in solving the SWEs is a critical criterion for assessing the effectiveness and robustness of numerical solution methods.

In this study, we adopt the parameters of the Earth for the SWEs on a rotating sphere. The initial conditions for the geopotential height and velocity fields are generated using Gaussian random fields, with parameters consistent with Bonev et al.^16^: mean initial layer depth $\varphi_{avg} = 10^{3} \,{\text{g}}$ with a standard deviation $\varphi_{std} = 120{\text{g}}$, mean initial velocity is zero with a standard deviation of $0.2\varphi_{avg}$, where $g \approx 9.81\, {\text{m}}/{\text{s}}^{2}$ is the acceleration of gravity.

After establishing the parameters and initial values for SWEs, a classical spectral solver^16,26^ is employed to generate the numerical solutions with a spatial resolution of 128 × 256 and time steps of 60 s. The training dataset is generated from 24 initial conditions, while the testing dataset is generated from 8 initial conditions. Each initial condition is simulated for a duration of 240 h; however, the first 48 h are excluded to address the spin-up problem. Solutions are recorded on an hourly basis. The solutions from the previous hour serve as inputs for the model, while the solutions at the next hour are utilized as labels.

We allocated 20% of the training data as the validation data and subsequently trained the U-Net^27^, FourCastNet^7^, SFNO^16^, and SMgNO models on the remaining 80% of the training data. The primary hyperparameters of these models are presented in Table S1 in the supplementary information. The training process utilizes a batch size of 16, with an initial learning rate set at 1.0 × 10^–3^, which decreases to 2.0 × 10^–5^ through cosine decay at the end of the training. The loss function employed is the weighted mean relative $\mathcal{L}2$ norm loss on the sphere, which is as follows^16^:

9 $$ {\mathcal{L}}2\left[ {F_{\vartheta } \left[ {u_{n} } \right],u_{n + 1} } \right] = \frac{1}{3}\mathop \sum \limits_{{c \in {\text{channels}} }} \left( {\frac{{\mathop \sum \nolimits_{{i \in {\text{ grid }}}} \,w_{i} \left| {F_{\vartheta } \left[ {u_{n} } \right]\left( {x_{i} } \right) - u_{n + 1} \left( {x_{i} } \right)} \right|^{2} }}{{\mathop \sum \nolimits_{{i \in {\text{ grid }}}} \,w_{i} \left| {u_{n + 1} \left( {x_{i} } \right)} \right|^{2} }}} \right)^{\frac{1}{2}} $$

where ${F}_{\vartheta }\left[{u}_{n}\right]$ is the predicted solutions and ${u}_{n+1}$ is the ground truth, ${w}_{i}$ is the product of the Jacobian $\text{sin}{\lambda }_{i}$ and the quadrature weights.

All fields (geopotential height and velocity components) were standardized using z-score normalization before training, where channel-wise means and standard deviations are calculated from the training set. Each model was trained for 50 epochs using a consistent training strategy (refer to Table S2 in the supplementary information for details), and the optimal weights were saved based on the validation data. Subsequently, the performance of each model was evaluated using the testing data. All experiments were conducted on a single Nvidia GeForce RTX 4090 GPU with 24 GB of memory.

### Data and experiment of global weather forecasting

The dataset utilized for autoregressive medium-range global weather forecasting is WeatherBench^28^, which is publicly available at https://github.com/pangeo-data/WeatherBench. WeatherBench contains regirded ERA5^29^ data from 1979 to 2018, with an hourly temporal resolution. It offers three spatial resolutions: 5.625° (32 × 64 grid points), 2.8125° (64 × 128 grid points), and 1.40525° (128 × 256 grid points). Given the constraints of our computational resources, the 5.625° spatial resolution was selected. Following prior studies, a time resolution of 6 h was adopted for the autoregressive forecasts.

Data from 1979 to 2015 was utilized as the training set, and data from 2016 was selected as the validation set. The out-of-sample data from 2017 to 2018 is employed as the testing set. This study incorporates 22 variables for autoregressive forecasting, which include 10U, 10V, T2M, U1000, V1000, Z1000, U850, V850, Z850, T850, RH850, U500, V500, Z500, T500, RH500, U250, V250, Z250, T250, T100, and Z50, respectively. The abbreviations and their corresponding descriptions are provided in Table S3 (see the supplementary information). Furthermore, the model input comprises two constant fields: the land-sea mask and the orography. The data was preprocessed using z-score normalization before training, and the evaluation metrics were calculated after de-normalization.

The models were developed using the PyTorch framework^30^, and the training workflow was provided by ClimaX^31^. The training procedure is similar to FourCastNet^7^, which consists pre-training steps and fine-tuning steps. During the pre-training steps, we employ supervised training to predict a single time step in the training dataset. In the fine-tuning steps, we start from the previously best pre-trained model and optimize the model to predict three time steps. The loss function utilized in this experiment is the latitude-weighted mean squared error (MSE), which is defined as follows:

10 $$ MSE\left[ {F_{\vartheta } \left[ {u_{n} } \right],u_{n + 1} } \right] = \frac{1}{C \times H \times W}\mathop \sum \limits_{c = 1}^{C} \,\mathop \sum \limits_{i = 1}^{H} \,\mathop \sum \limits_{j = 1}^{W} w_{i} \left( {F_{\vartheta } \left[ {u_{n} } \right]\left( {x_{c, i, j} } \right) - u_{n + 1} \left( {x_{c, i, j} } \right)} \right)^{2} $$

where $C, H, W$ are the number of channels, grid points in latitude, grid points in longitude, respectively. ${F}_{\vartheta }\left[{u}_{n}\right]\left({x}_{c, i, j}\right)$ and ${u}_{n+1}\left({x}_{c, i, j}\right)$ are the predicted and ground truth at time step of $n+1$. ${w}_{i}$ is the weighting factor for the latitude, which is calculated as follows:

11 $$ w_{i} = \frac{{\cos \left( {{\text{lat}}\left( i \right)} \right)}}{{\frac{1}{H}\mathop \sum \nolimits_{i}^{H} \,\cos \left( {{\text{lat}}\left( i \right)} \right)}} $$

where $\text{cos}$ is the cosine function. Both the pre-training and fine-tuning steps utilize the Brain Floating Point half-precision format and the AdamW^32,33^ optimizer with parameters ${\beta }_{1} = 0.9, {\beta }_{2} = 0.99$ and a weight decay of 1.0 × 10^–5^.

The pre-training process was conducted over 100 epochs with a batch size of 80 and the initial learning rate was 2.0 × 10^–4^, accompanied by a linear warmup schedule for 6 epochs, followed by a cosine-annealing schedule^34^ for the subsequent 94 epochs. The fine-tuning process was carried out over 10 epochs with a batch size of 32 and the initial learning rate was 2.0 × 10^–4^, accompanied by a linear warmup schedule for 1 epochs, also followed by a cosine-annealing schedule^34^ for the remaining epochs. The best weights for each model were saved according to the latitude-weighted root mean square error (RMSE) on the validation data. The evaluation metrics on the testing data, namely latitude-weighted RMSE and anomaly correlation coefficient (ACC), which were calculated as follows^5,28^:

12 $$ {\text{RMSE}} = \frac{1}{{N_{{\text{forecasts }}} }}\mathop \sum \limits_{n}^{{N_{{\text{forecasts }}} }} \sqrt {\frac{1}{{N_{{\text{lat }}} \,N_{{\text{lon }}} }}\mathop \sum \limits_{i}^{{N_{{\text{lat }}} }} \,\mathop \sum \limits_{j}^{{N_{{\text{lon }}} }} \,w_{i} \left( {f_{n,i,j} - t_{n,i,j} } \right)^{2} } $$

13 $$ {\text{ACC}} = \frac{{\mathop \sum \nolimits_{n,i,j} \,w_{i} f_{n,i,j}^{\prime } \,t_{n,i,j}^{\prime } }}{{\sqrt {\mathop \sum \nolimits_{n,i,j} \,w_{i} f_{n,i,j}^{\prime 2} \mathop \sum \nolimits_{n,i,j} \,w_{i} t_{n,i,j}^{\prime 2} } }} $$

where $f$ is the model forecast and $t$ is the ERA5 truth, ${w}_{i}$ is the latitude weighting factor for the latitude at the $i$-th latitude index, the prime ′ denotes the difference to the climatology and the climatology is defined as ${\text{climatology}}_{i,j}=\frac{1}{{N}_{\text{time }}}\sum {t}_{i,j}$. The primary hyperparameters utilized in this study are presented in Table S1. All models were trained on a single NVIDIA GeForce RTX 4090 GPU, employing a consistent training strategy. For further details, please refer to Table S2 in the supplementary information.

## Results

### Spherical shallow water equations

**Fig. 2:**
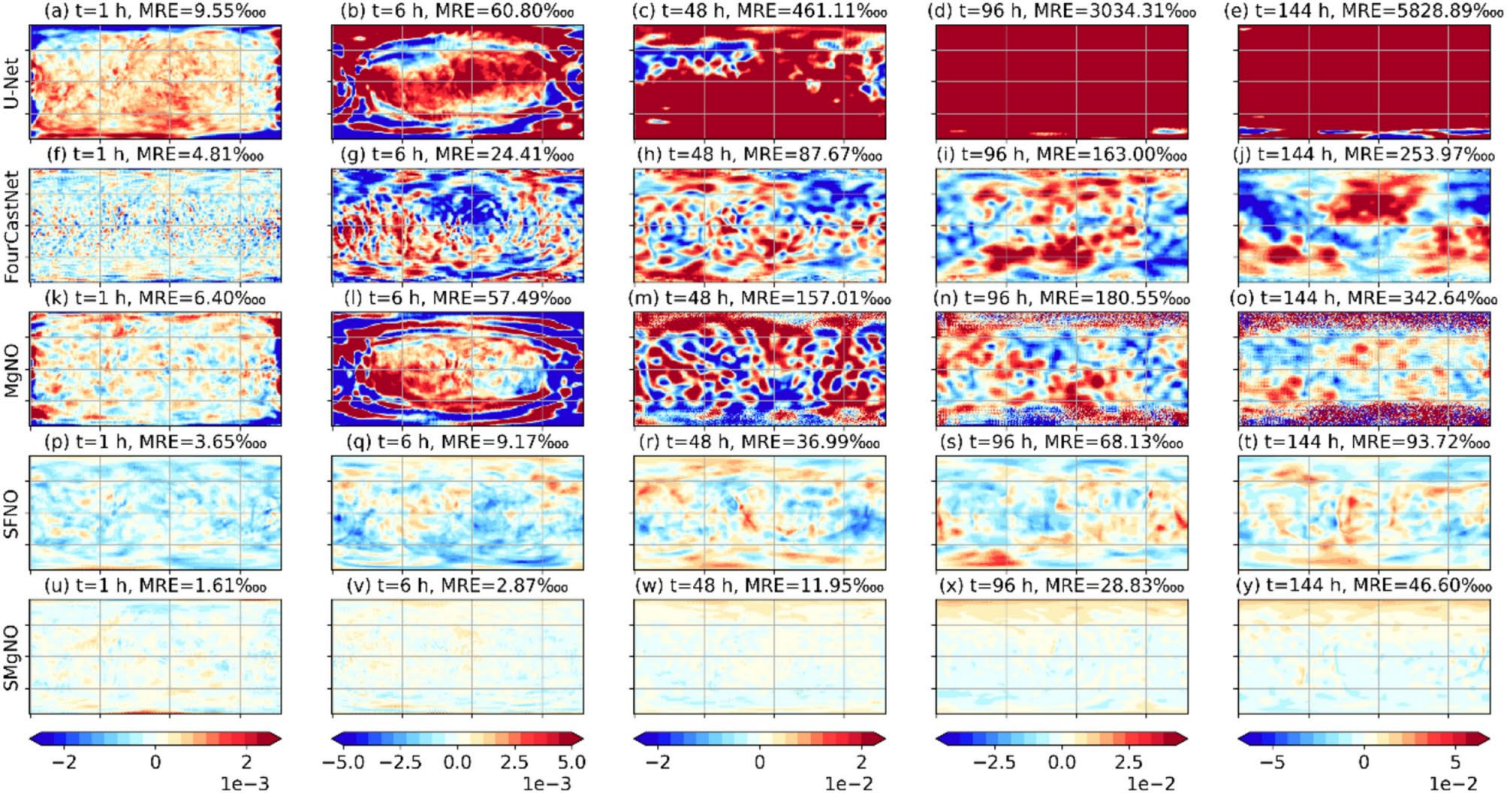
Spatial distribution of relative errors for geopotential height in spherical shallow water equations. The smaller the absolute value, the better the performance. Columns from left to right corresponding to 1, 6, 48, 96 and 144 autoregressive steps respectively. Rows from top to bottom represent the U-Net, FourCastNet, MgNO, SFNO, and SMgNO respectively. The mean relative error (MRE) for each example was given in the subFigure title. This Figure was created using Matplotlib library version 3.8.4 (https://matplotlib.org/) on Python 3.10.13 (https://www.python.org).

We begin by experimentally demonstrating that conventional convolution models cause distortions when processing spherical data. The widely used U-Net^27^, FourCastNet^7^ and MgNO^17^ were selected as the baseline models for conventional convolution, while SFNO^16^ was chosen as the baseline model for spherical convolution. As illustrated in Fig. 2a–e, the relative errors of geopotential height forecasted by U-Net are notable near the poles and along the east–west boundary, even at the first iteration. The primary contributors to these errors are the distortions at the poles and the zero-padding at the boundaries. These errors will propagate from the poles to mid and low latitudes and from the east–west boundary to the interior regions across autoregressive steps, and gradually affecting the entire domain. Despite performing better than U-Net, MgNO has comparable difficulties (see Fig. 2k–l). Furthermore, because MgNO uses transposed convolution as the upsampling method instead of interpolation, it suffers from checkerboard distortions^21^. These checkerboard artifacts have more severe effects as the number of autoregressive steps increases, which exacerbates the polar distortion (see Fig. 2m–o). The discrete Fourier transform (DFT) in FourCastNet ensures continuity at the east–west boundary, making it exhibit lower relative errors than U-Net and MgNO. However, due to the implicit periodicity in the meridian direction and the flat assumption of the DFT, FourCastNet still suffers data distortion, leading to a rapid increase in relative errors across autoregressive steps (see Figs. 2f–j and S1 in the supplementary information). To mitigate these distortions, SFNO incorporated SHT into the data-driven model, which maintains the continuity of the east–west boundary and reduces spherical distortions, thereby significantly enhancing stability and performance (see Fig. 2p–t). The SMgNO, similar to SFNO, which employs CSHFs to alleviate spherical distortions, also demonstrates stable autoregressive forecasts (Fig. 2u–y).

Despite the improvements made by SFNO, it requires more computational resources than FourCastNet. To quantify these computational demands, we calculated the number of floating-point operations (FLOPs) for each model using the calflop^35^ package and measured peak GPU memory consumption during training via a function in PyTorch^30^. As indicated in Table 1, when the number of parameters is similar, SFNO demands approximately 63.43% more FLOPs for forward propagation than FourCastNet. Furthermore, SFNO requires 2.32 times more GPU memory than FourCastNet during training. The MgNO, on the other hand, has the lowest number of FLOPs and requires the lowest GPU memory for training. Due to the integration of CSHFs, the computational costs of SMgNO are slightly higher than MgNO, but greatly smaller than SFNO which benefits from a multigrid framework.

**Table 1:** The number of model parameters and computational costs for spherical shallow water equations and medium-range global weather forecasting.

Models	Spherical shallow water equations			Medium-range global weather forecasting		
	Params (M)	FLOPs (G)	Peak GPU memory (MB)	Params (M)	FLOPs (G)	Peak GPU memory (MB)
U-Net	34.53	65.44	768.87	–	–	–
FourCastNet	9.78	35.19	435.49	23.59	**1.47**	559.18
MgNO	8.99	**4.26**	**210.33**	–	–	–
SFNO	9.97	57.51	1008.31	21.07	38.90	713.03
SMgNO	**7.94**	8.46	350.43	**20.63**	3.84	**492.68**

In the table, “FLOPs” refer to the number of floating-point operations required for a model’s forward propagation; peak GPU memory was measured when the batch size was set to 1 during training; “–” indicates not applicable. The peak GPU memory of the medium-range global weather forecasting shown in the table is for the pre-training. For clarity, the smallest value is in bold and the second smaller is underlined.

**Fig. 3:**
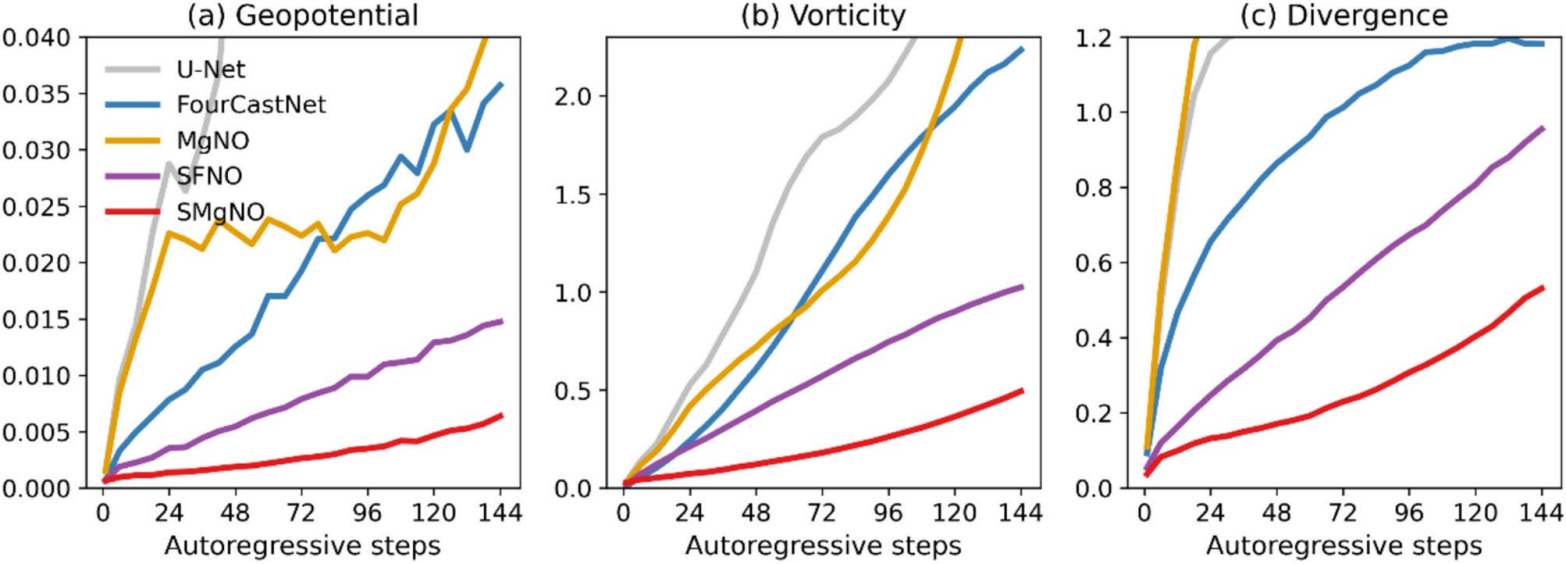
Weighted mean relative $\mathcal{L}2$ norm losses for the shallow water equations on the rotating sphere at a spatial resolution of 128 × 256 and a temporal resolution of 1 h. In the  Figure, the x-axis represents autoregressive steps (1 to 144) i.e. lead times (1 h to 144 h), and the y-axis is the values of weighted mean relative $\mathcal{L}2$ norm losses. The lower the weighted mean relative losses the better the performance. (**a**) mean relative losses of geopotential height; (**b**) mean relative losses of vorticity; (**c**) mean relative losses of divergence.

To further illustrate the performance of the model, Fig. 3 presents the weighted mean relative losses for solving the SWEs on the rotating sphere at a temporal resolution of one hour and a spatial resolution of 128 × 256. As depicted, the losses of U-Net accumulate rapidly across autoregressive steps. The MgNO outperforms the U-Net, even has a slightly lower relative losses than the FourCastNet in the intermediate autoregressive steps. However, it is still difficult to control the accumulation of errors for MgNO. Generally speaking, the FourCastNet performs better than the U-Net and the MgNO, but does not match the performance of the SFNO. The proposed SMgNO combines the advantages of SFNO and MgNO, it mitigates spherical distortion (Fig. 2u–y) and has the best autoregressive performance (as depicted in Fig. 3) while requiring low computational costs (see Table 1).

### Ablation experiments

**Fig. 4:**
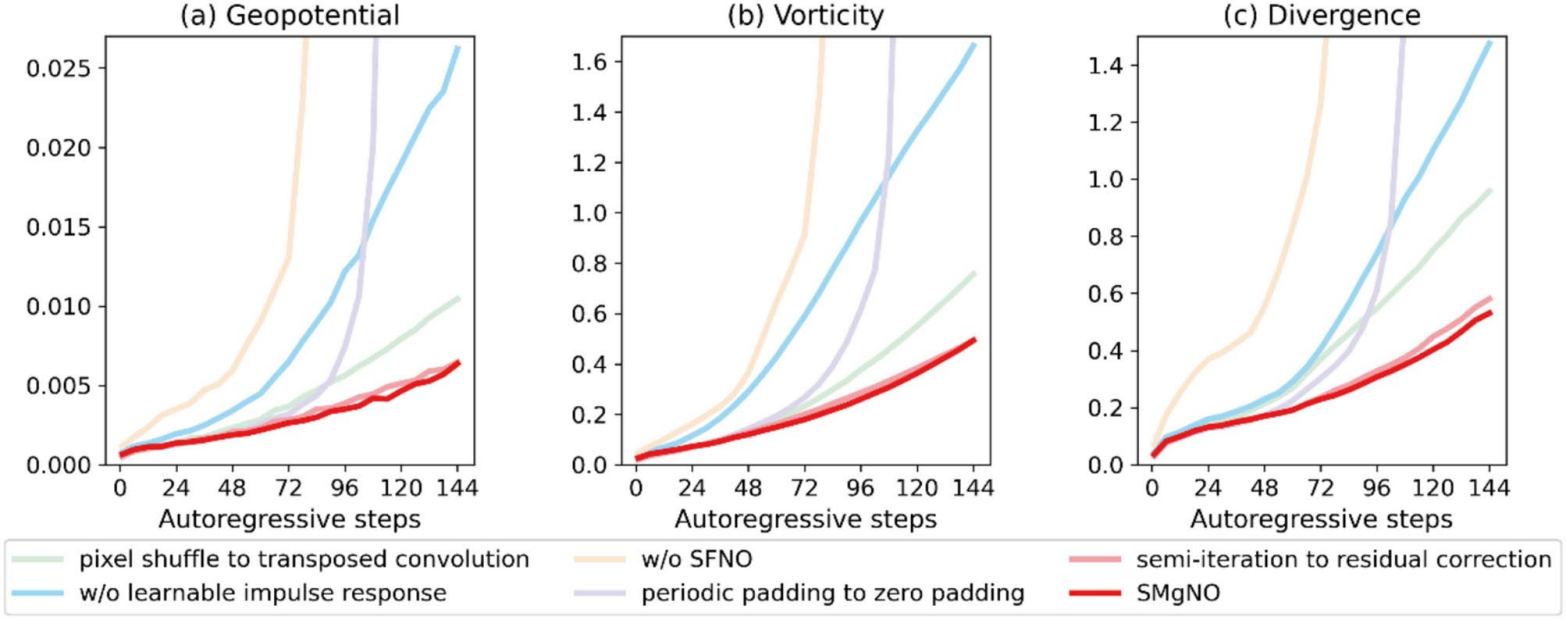
Weighted mean relative $\mathcal{L}2$ norm losses for ablation experiments. In the Figure, the x-axis represents autoregressive steps (1 to 144) i.e. lead times (1 h to 144 h), and the y-axis is the values of weighted mean relative $\mathcal{L}2$ norm losses. The lower the weighted mean relative losses the better the performance. (**a**) mean relative losses of geopotential height; (**b**) mean relative losses of vorticity; (**c**) mean relative losses of divergence. In the Figure “w/o” is short for “without”.

To validate the contributions of individual components within our architecture, we conducted a systematic series of ablation studies. By progressively removing or modifying key modules and evaluating their impacts on relative $\mathcal{L}2$ norm losses, we quantified the role of each component. The results, summarized in Fig. 4, show that incorporating the SFNO in the coarse grid significantly enhances the stability and accuracy of autoregressive forecasts while greatly increasing the number of model parameters and FLOPs (see Table S4 in the supplementary information). The second improvement is attributed to periodic padding in the longitudinal direction. When the number of autoregressive steps is small, the error introduced by zero padding is not evident; however, as the number of autoregressive steps increases, the errors become substantial. Guarantee the continuity of the east–west boundary through periodic padding in the longitudinal direction prevents the outbreak of errors. Moreover, altering the padding mode does not increase the number of parameters and FLOPs. Another efficient improvement is made by the learnable pulse at the poles, which only slightly increases the number of model parameters and FLOPs. This indicates that the truncation error associated with SHT does influence the stability and accuracy of autoregressive forecasts. Substituting the transposed convolution with pixel shuffle does reduce the relative errors of muti-step autoregression. Furthermore, using the semi-iteration smoothing slightly improves the autoregressive performance.

### Medium-range global weather forecasting

For medium-range global weather forecasting, we train the FourCastNet^7^, the SFNO^16^, and the proposed SMgNO model utilizing data from WeatherBench^28^ and employing a consistent training methodology. Each model has approximately 21 million parameters (see Table 1 for more information), and their performance is evaluated in comparison to the IFS T42 model using the testing dataset.

**Fig. 5:**
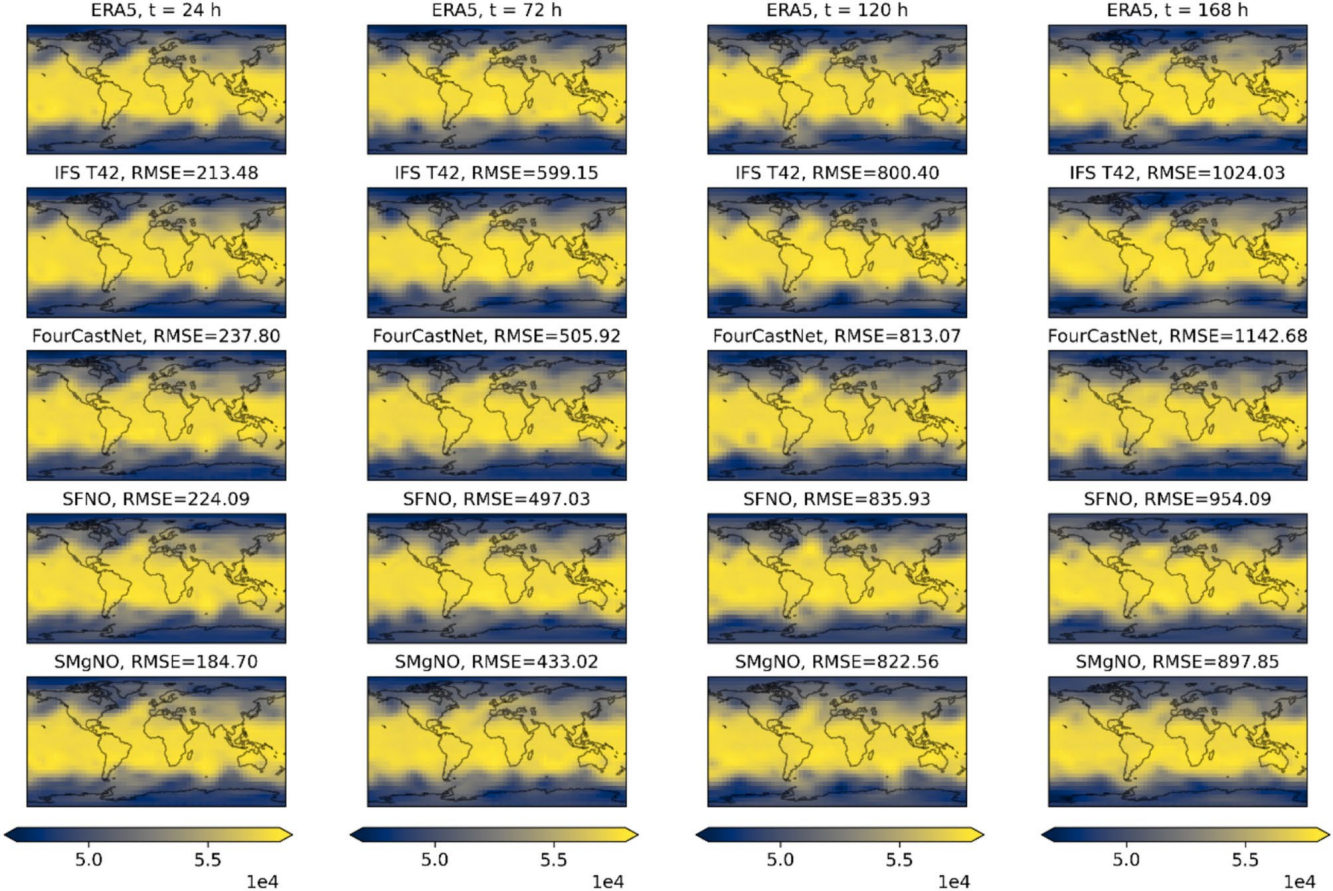
Visualization of forecast results for geopotential (m^2^/s^2^) at the 500 hPa pressure level. Columns from left to right correspond to 1 day, 3 days, 5 days, and 7 days of lead time, i.e., 4, 12, 20, and 28 autoregressive steps, respectively. Rows from top to bottom represent the ERA5 (ground truth), IFS T42, Fourcastnet, SFNO, and SMgNO models. In the Figure, RMSE is the abbreviation of root mean square error. For all cases, the input time is 00:00 UTC on 12 March 2017, and the spatial resolution is 5.625° × 5.625°. This  Figure was created using Matplotlib library version 3.8.4 (https://matplotlib.org/) and Cartopy library version 0.23.0 (https://scitools.org.uk/cartopy) on Python 3.10.13 (https://www.python.org), with coastline data from Natural Earth public domain datasets (https://www.naturalearthdata.com/).

Figure 5 presents the geopotential at the 500 hPa pressure level predicted by the SMgNO and baseline models, which are all initialized at 2017-03-012T00:00:00 UTC. It can be seen that with the increase of autoregressive steps, the RMSE increases gradually. FourCastNet is comparable to that of the SFNO at the initial autoregression but falls behind as the number of autoregressive steps increases. The DFT employed in FourCastNet introduces distortions near the poles, which adversely affect the accuracy and stability of autoregressive forecasts. Therefore, FourCastNet needs to tune the parameters or improve the autoregressive strategy. SFNO incorporated the SHT in data-driven models, mitigating the effects of spherical data distortion and achieving superior autoregressive performance compared to FourCastNet. Compared with these baseline models, the proposed SMgNO model has the best autoregressive performance. The temperature at the 850 hPa pressure level illustrated in Fig. S2 (in the supplementary information) shows similar results.

**Fig. 6:**
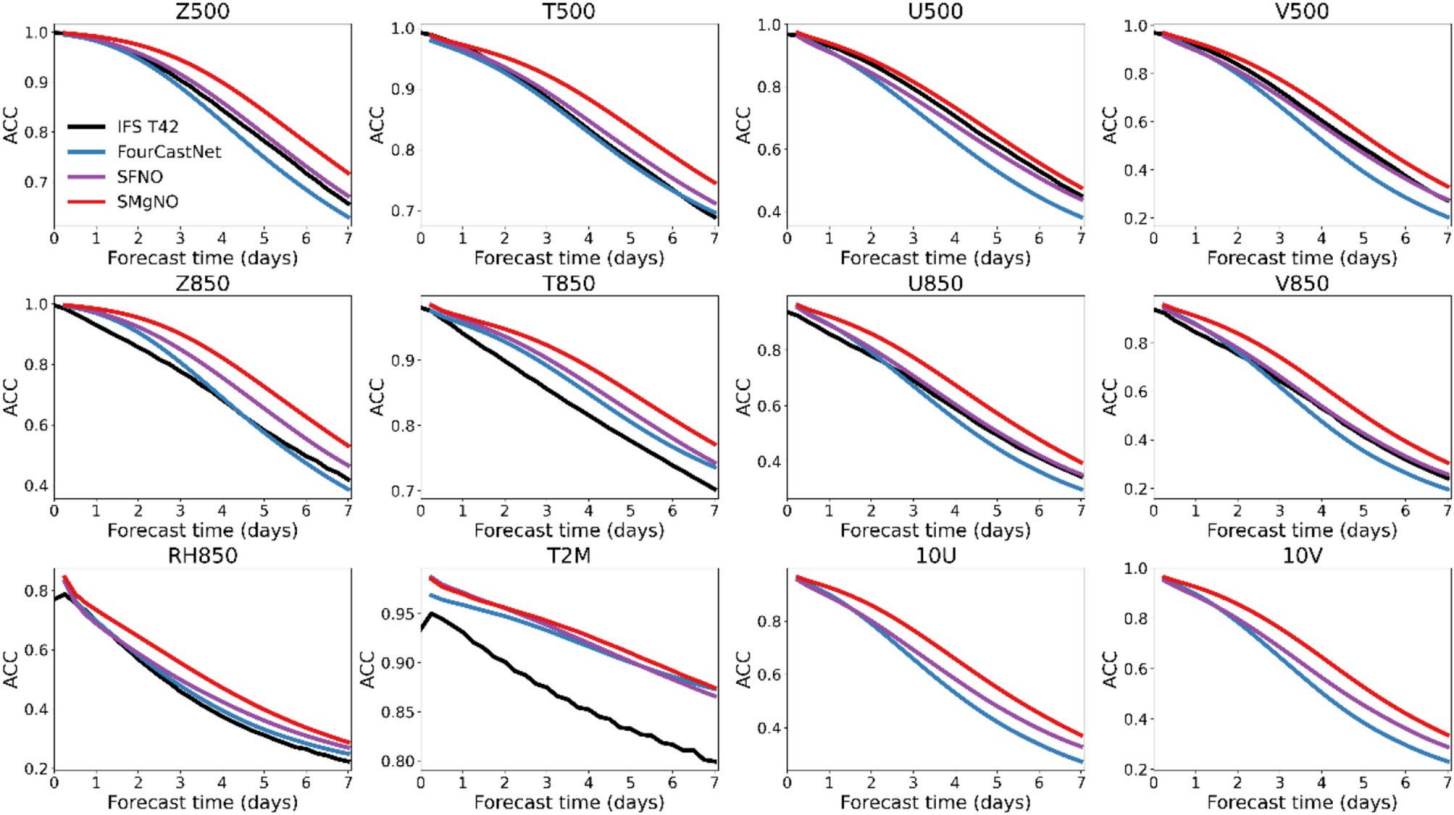
Globally averaged latitude-weighted anomaly correlation coefficient (ACC) of IFS T42 (black lines), FourCastNet (blue lines), SFNO (purple lines), and SMgNO (red lines) for 3 surface variables, 4 upper-air variables at 500 hPa pressure level, and 5 upper-air variables at 850 hPa pressure level with spatial resolution of 5.625° × 5.625° in 7 days forecasts using testing data from 2017 to 2018. In the Figure, the x-axis represents lead times, and the y-axis is the values of globally averaged latitude-weighted ACC. The higher the value of ACC, the better the performance. IFS T42 were not available for 10U and 10 V.

Figure 6 illustrates the globally-averaged latitude-weighted ACC of various models across different lead times of three surface variables (T2M, U10, and V10), four upper-air variables (Z500, T500, U500, and V500) at the 500 hPa pressure level, and five upper-air variables (Z850, T850, U850, V850, and RH850) at the 850 hPa pressure level. The ACC of FourCastNet is comparable to that of the other models during the early stages of autoregression. However, it experiences a rapid decline as the number of autoregressive steps increases. For instance, the geopotential at the 850 hPa pressure level predicted by FourCastNet initially surpasses that of the IFS but falls behind when the lead time exceeds four days (consistent with the findings of Pathak, Subramanian^7^). Despite the SFNO having superior performance compared to FourCastNet, it incurs higher computational costs (refer to Table 1), and the accuracy of the velocity field at the 500 hPa pressure level remains inferior to that of the IFS T42. The proposed SMgNO not only outperforms both the IFS T42 and SFNO but also reduces computational expenses. For instance, the ACC of SMgMO for geopotential height with a 7 days lead time increases by 9.31% and 6.83% compared to IFS T42 and SFNO, respectively. Meanwhile, SMgNO requires only 10% FLOPs of SFNO for forward propagation and 30.90% less GPU memory consumption during training than SFNO (see Table 1 for details). Furthermore, SMgNO even requires 11.89% less GPU memory than FourCastNet during training, despite having a comparable number of model parameters (see Table 1). Figure S3 in the supplementary information presents the globally-averaged latitude-weighted RMSE, further demonstrating that SMgNO has better autoregressive forecasts than the bassline models.

## Discussion

Recent advancements in data-driven global weather forecasting systems^1,2,4,8^ have demonstrated superior deterministic forecasting capabilities compared to the ECMWF’s IFS^36^. However, conventional convolution- and Transformer-based models introduce geometric distortions when handling spherical data, causing instability in autoregressive forecasts. SFNO can alleviate such distortions but needs expensive computational costs. To address these challenges, we propose SMgNO, which integrates convolution based on spherical harmonic functions (CSHFs) to preserve geometric fidelity and employs a multigrid framework to reduce computational costs. Experiments on spherical SWEs and medium-range global weather forecasting demonstrate the superiority of SMgNO. For 500 hPa geopotential height forecasting with a 7 days lead time, SMgNO achieves a 9.31% and 6.83% improvement in ACC over IFS T42 and SFNO, respectively. Notably, these performance gains are attained with substantially reduced computational demands, for which SMgNO requires only 10% FLOPs of SFNO for forward propagation and 30.90% less GPU memory consumption during training than SFNO.

Despite the promising performance of SMgNO, some limitations remain. First, the model is trained through a mean squared error (or $\mathcal{L}2$) loss function, which may smooth the information of fine scales through a “double penalty” effect—where biases in the location of phenomena are penalized twice. Second, although SMgNO requires fewer computational resources than SFNO, the introduction of CSHFs inevitably increases computational costs compared to MgNO.

In future work, we will develop a spectral or multi-scale loss function to alleviate the double-penalty effect and improve the forecast performance of fine scales. Furthermore, we will reduce computational overhead by implementing spherical convolutions with Hierarchical Equal Area isoLatitude Pixelization (HEALPix)^37^ discretization and evaluate its performance on different resolutions. Finally, we will explore how to balance the computational cost and forecast accuracy of the proposed method in operational applications.

## Supplementary Information

Supplementary Information.

## Data Availability

Data for Shallow Water Equations on the rotating sphere is generated through the code at https://github.com/NVIDIA/torch-harmonics. WeatherBench is publicly available at https://github.com/pangeo-data/WeatherBench.
