# Supplementary material for: Spherical multigrid neural operator for improving autoregressive global weather forecasting
Source: Sci Rep. 2025 Apr 4;15:11522. doi: 10.1038/s41598-025-96208-y (PMC11968892; doi:10.1038/s41598-025-96208-y)
Supplement: Supplementary file 1 — Supplementary Information. [file 41598_2025_96208_MOESM1_ESM.docx]

**Supplementary Information for “Spherical Multigrid Neural Operator for Improving Autoregressive Global Weather Forecasting”**

Yifan Hu^1, 2^, Fukang Yin^2, *^, Weimin Zhang^2, *^, Kaijun Ren^2^, Junqiang Song^2^，Kefeng Deng^2^, and Di Zhang^2^

^1^College of Computer Science and Technology, National University of Defense Technology, Changsha, P.R. China, 410073

^2^College of Meteorology and Oceanography, National University of Defense Technology, Changsha, P.R. China, 410073

*Corresponding author(s). E-mail: [yinfukang@nudt.edu.cn](mailto:yinfukang@nudt.edu.cn) (F, Yin) ; [weiminzhang@nudt.edu.cn](mailto:weiminzhang@nudt.edu.cn) (W, Zhang) .

## Contents of this file

Supplementary Texts

Supplementary Tables S1 to S4

Supplementary Figures S1 to S3

## Supplementary Texts

The pseudo-code of multigrid method is as follows.

| **Algorithm S1** $MG\left( f \right)$. The multigrid method with V-cycle for the solution of the linear system $Au = f$. |
| --- |
| 1. Initialization: Set $f^{1} = input\left( f \right)$, $u^{1, 0} = 0$ 2. Smoothing and restriction from fine to coarse level: 3. for $l = 1:L$ do 4. Pre-smoothing: 5. for $i = 1 : v_{l}$ do 6. $\tilde{u}^{l, i} = u^{l, i - 1} + S^{l,i - 1}(f^{l} - A^{l}u^{l, i - 1})$ 7. $u^{l, i} = \tilde{u}^{l, i} + S^{l,i}(f^{l} - A^{l}\tilde{u}^{l, i})$ 8. end for 9. if $l < L$: 10. Restriction from fine to coarse level and set initial guess:   $u^{l + 1,0} = 0$, $f^{l + 1}=R_{l}^{l + 1}(f^{l}-A^{l}u^{l,v_{l}})$   1. end if 2. end for 3. Solve $A^{L}u^{L,v_{l}}=f^{L}$ through numerical solver 4. for $l = L - 1 : 1$do 5. Prolongation from coarse to fine level: $x^{l, 0}={u^{l,v_{l}}+P}_{l + 1}^{l}(u^{l + 1,v_{l}})$ 6. Post-smoothing: 7. for $i = 1 : v_{l}$ do 8. $\tilde{x}^{l, i}=x^{l, i - 1}+S^{l,i-1}(f^{l}-A^{l}x^{l, i - 1})$ 9. $x^{l, i}=\tilde{x}^{l, i}+S^{l,i}(f^{l}-A^{l}\tilde{x}^{l, i})$ 10. end for 11. end for 12. Output ${u=x}^{1, v_{l}}$ |

## Supplementary Tables

**Table S1.** The hyperparameters of different models. In the table, “--" indicates not applicable.

| Hyper-parameter | spherical shallow water equations | | | medium-range global weather forecasting | | |
| --- | --- | --- | --- | --- | --- | --- |
|  | FourCastNet | SFNO | SMgNO | FourCastNet | SFNO | SMgNO |
| patch size | 4 × 4 | -- | -- | 8 × 8 | -- | -- |
| number of blocks | 16 | -- | -- | 8 | -- | -- |
| depth or layers | 4 | 4 | 4 | 8 | 4 | 4 |
| MLP ratio | 4 | 2 | 2 | 2 | 2 | 2 |
| embedding dimension | 512 | 256 | 256 | 768 | 928 | 512 |
| activation function | GELU | GELU | GELU | GELU | GELU | GELU |
| number of levels | -- | -- | 3 | -- | -- | 3 |
| channels per level | -- | -- | 32 | -- | -- | 192 |

**Table S2.** Training configurations for different experiments. All models were trained using the same training strategy to ensure a fair comparison. In the table, “LR” is short for “learning rate”.

| Experiments | | Losses | Initial LR | LR scheduler | Optimizer | Batch size | Epochs |
| --- | --- | --- | --- | --- | --- | --- | --- |
| spherical shallow water equations | | weighted mean relative $\mathcal{L}2$ norm | 10^−3^ | cosine annealing | Adam | 16 | 50 |
| global weather forecasting | pre-training | latitude-weighted $\mathcal{L}2$ | 2.0 × 10^-4^ | linear warmup with cosine annealing | AdamW | 80 | 100 |
|  | fine-tuning |  |  |  | AdamW | 32 | 10 |

**Table S3.** The abbreviations and their descriptions for different variables.

| Abbreviation | Description |
| --- | --- |
| 10U | zonal wind velocity at 10m from the surface |
| 10V | meridional wind velocity at 10m from the surface |
| T2M | temperature at 2m from the surface |
| U--- | zonal wind velocity at pressure level --- |
| V--- | meridional wind velocity at pressure level --- |
| Z--- | geopotential at pressure level --- |
| T--- | temperature at pressure level --- |
| RH--- | relative humidity at pressure level --- |

**Table S4.** The number of model parameters and floating-point operations (FLOPs) for ablation experiments. In the table“w/o”is short for “without”.

| Model | Params (M) | FLOPs (G) |
| --- | --- | --- |
| SMgNO: pixel shuffle to transposed convolution | 7.90 | 8.04 |
| SMgNO: w/o learnable impulse response | 7.94 | 8.45 |
| SMgNO: w/o SFNO | 5.88 | 1.44 |
| SMgNO: periodic padding to zero padding | 7.94 | 8.46 |
| SMgNO: semi-iteration to residual correction | 7.94 | 8.46 |

## Supplementary Figures


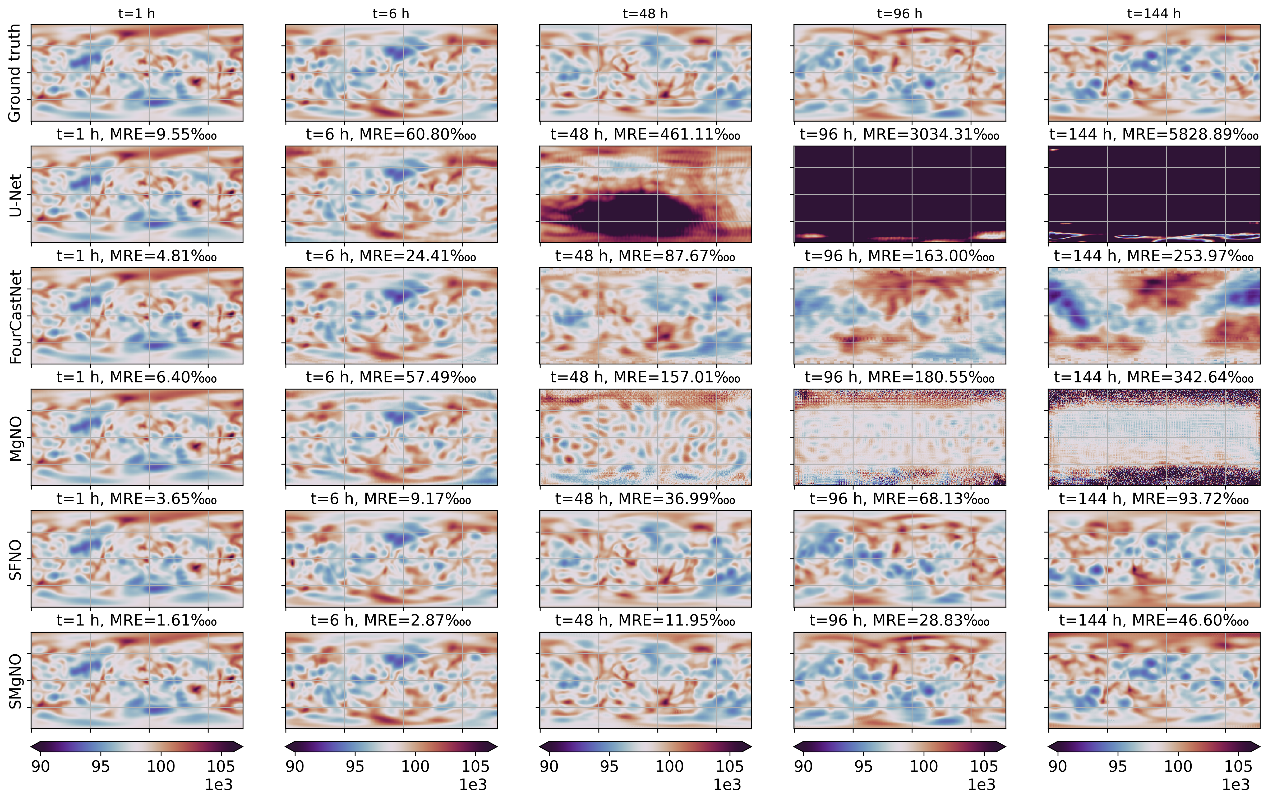


**Figure S1.** Visualization of geopotential (m^2^/s^2^) for spherical shallow water equations. Columns from left to right corresponding to 1, 6, 48, 96 and 144 autoregressive steps respectively. Rows from top to bottom represent the ground truth, U-Net, FourCastNet, MgNO, SFNO, and SMgNO respectively. The mean relative error (MRE) for each example was given in the subfigure title. The initial input fields are the same as the Figure 2. This figure was created using Matplotlib library version 3.8.4 (https://matplotlib.org/) on Python 3.10.13 (https://www.python.org).


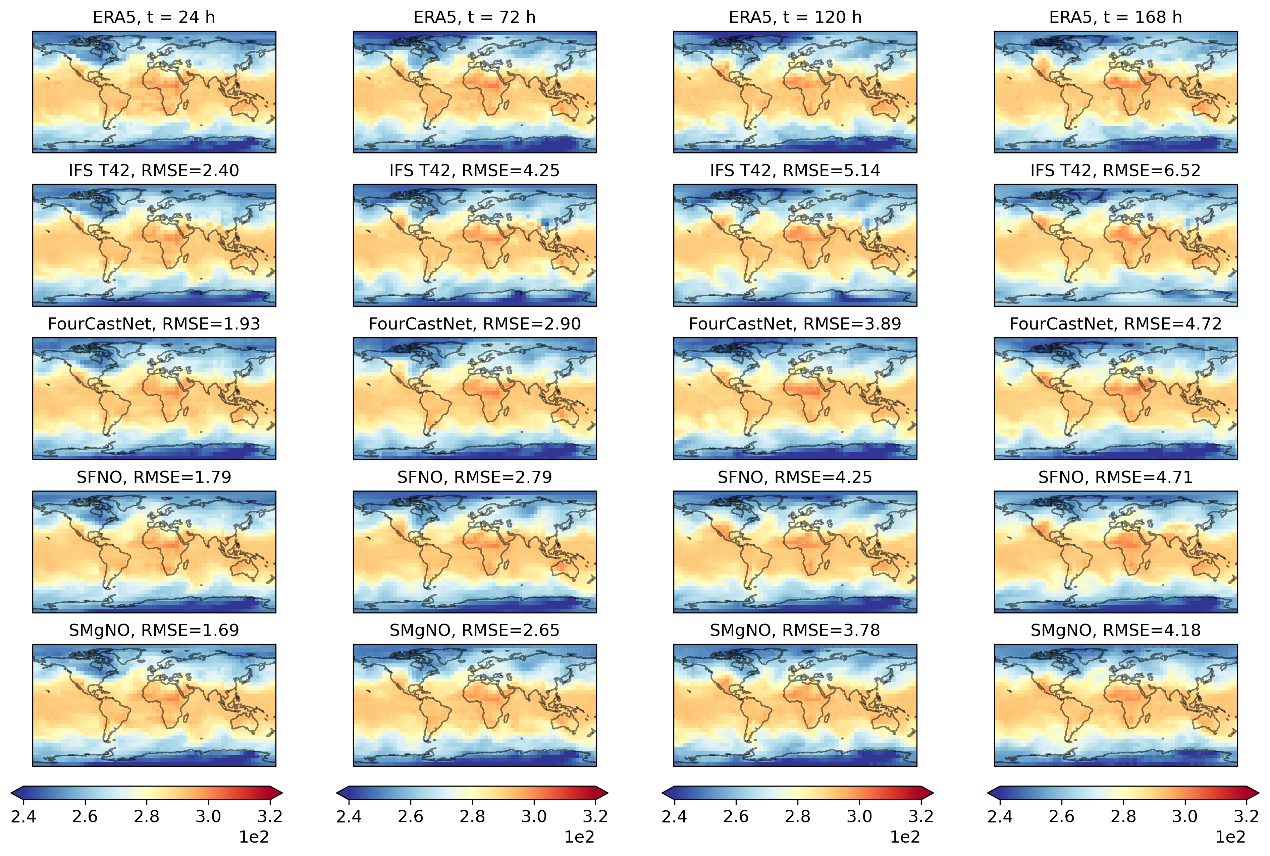


**Figure S2.** Visualization of forecast results for temperature (K) at the 850 hPa pressure level. Columns from left to right correspond to 1 day, 3 days, 5 days, and 7 days of lead time, i.e., 4, 12, 20, and 28 autoregressive steps, respectively. Rows from top to bottom represent the ERA5 (ground truth), IFS T42, FourCastNet, SFNO, and SMgNO models. In the figure, RMSE is the abbreviation of root mean square error. For all cases, the input time is 00:00 UTC on 12 March 2017, and the spatial resolution is 5.625° × 5.625°. This figure was created using Matplotlib library version 3.8.4 (https://matplotlib.org/) and Cartopy library version 0.23.0 (https://scitools.org.uk/cartopy) on Python 3.10.13 (https://www.python.org), with coastline data from Natural Earth public domain datasets (https://www.naturalearthdata.com/).


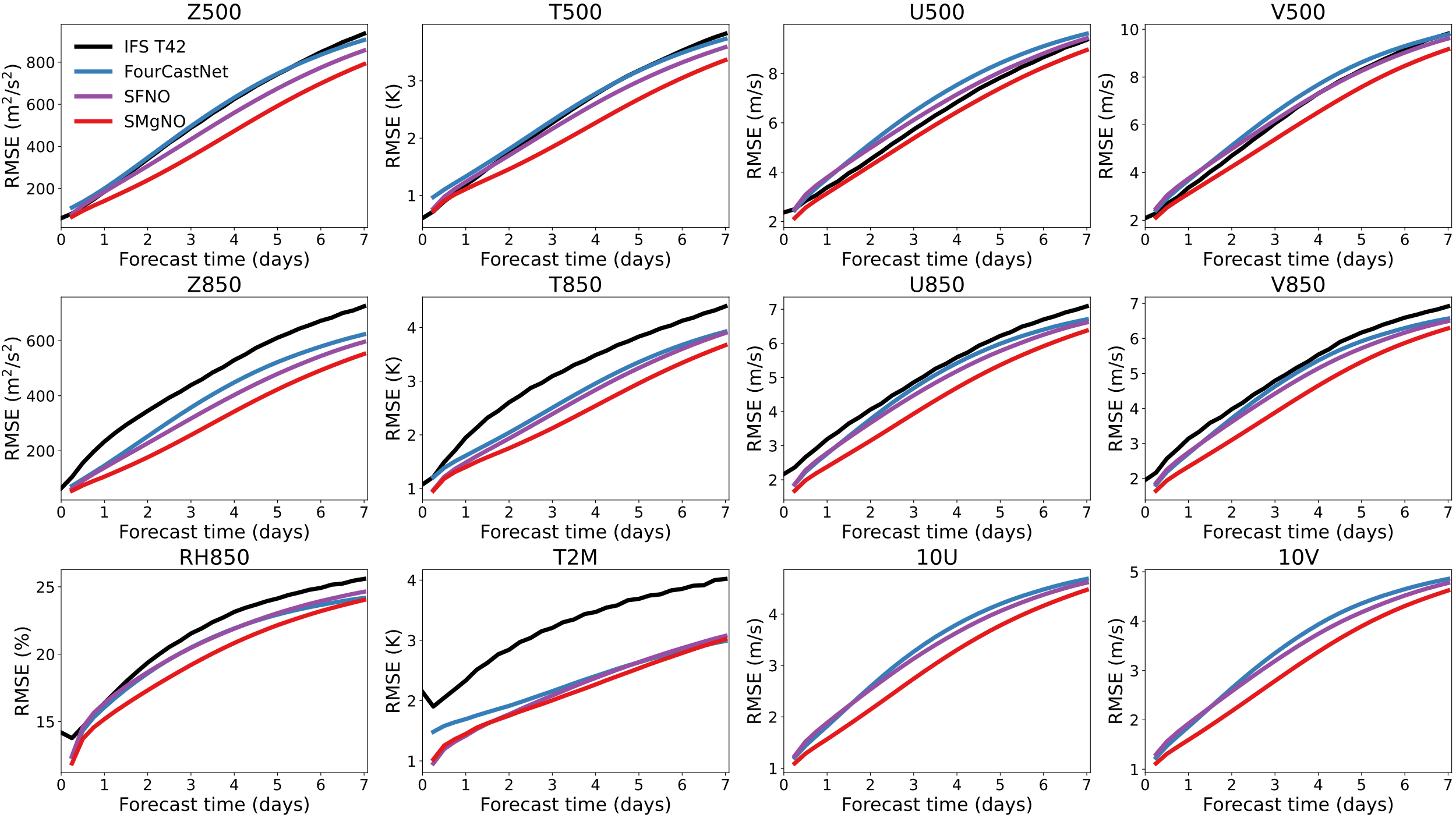


**Figure S3.** Globally averaged latitude-weighted root mean square error (RMSE) of IFS T42 (black lines), FourCastNet (blue lines), SFNO (purple lines), and SHNO (red lines) for 3 surface variables, 4 upper-air variables at 500 hPa pressure level, and 5 upper-air variables at 850 hPa pressure level with spatial resolution of 5.625° × 5.625° in 7 days forecasts using testing data from 2017 to 2018. In the figure, the x-axis represents lead times, and the y-axis is the values of globally averaged latitude-weighted RMSE. The lower the value of RMSE, the better the performance. IFS T42 were not available for 10U and 10V.
